# Stance modals in Chinese EFL learners’ monologue tasks: A corpus-based study

**DOI:** 10.1371/journal.pone.0347841

**Published:** 2026-04-24

**Authors:** Wei Wang, Haixiao Wang

**Affiliations:** Department of Applied Foreign Language Studies, Nanjing University, Nanjing, China; Bahir Dar University, ETHIOPIA

## Abstract

This study investigates how Chinese tertiary-level EFL learners used modals as linguistic devices for expressing stances in spoken English. Modals convey speakers’ commitment to propositions (epistemic stance) and intentions to affect reality (effective stance). While existing research has primarily focused on written registers and epistemic stances, this corpus-based study analyzes both stances in a national-level standardized speaking test, examining modals’ frequency, choices, and tonal variations during an opinion-giving task. Our findings reveal that learners commonly used epistemic and effective modals but leaned more toward effective modals than native speakers. Their speech exhibited assertiveness, marked by high use of *will*, *should*, and *must* while showing less reliance on *would* and *have to*. Moreover, *can* was often employed with conditional clauses/phrases to soften claims and used in rhetorical sentences to enhance assertiveness. These patterns could be attributable to the influence of the Chinese language, rhetorical norms, cultural influences, limited linguistic resources, and teaching support. Our findings add knowledge about modal stance in speaking tasks by revealing patterns of CEFLLs’ modal use during the period examined. These findings also offer implications for addressing learners’ reliance on certain modals.

## 1. Introduction

Stance refers to how writers or speakers express their feelings, attitudes, and judgments [1]. It concerns interactional positioning [2], involving the writers’ or speakers’ attitude towards different viewpoints and their alignment with the audience [3] and reflecting personal evaluation and sociocultural values [4,5].

Stance is realized through various linguistic devices, including modal verbs, evaluative adjectives, and audience pronouns. Among these, modals have been extensively studied, particularly epistemic modals (e.g., *may*, *might*), which signal the speaker’s commitment to the likelihood of a proposition [6–9]. Researchers’ dense interest in epistemic modals may stem from the predominance of this modal type in academic writing, where deontic modals (e.g., *must*, *should*), which express obligation or necessity, are infrequent ([1], p. 489). However, recent studies argue that both types contribute to stance-taking in speaking [10]. Effective stance [11], encompassing deontic and dynamic modals (e.g., *can*, *need*), explicitly positions speakers in the realization of events through persuasion or coercion (p. 414), complementing the epistemic focus on possibility and knowledge source.

The significance of deontic modals in EFL contexts is further underscored by learners’ tendency to invoke external authority through obligation markers [12,13]. Despite their low frequency in academic prose, deontic modals and multifunctional modals like *can* are critical in spoken discourse, where persuasion and appealing dominate [10]. The use of deontic modals is more accessible to L2 student writers than that of epistemic modals [14,15]. Even for published L2 writers, they tended to use more deontic modals than L1 English authors do [16]. Yet, prior research has predominantly analyzed written texts, neglecting the role of modals in oral tasks. This gap motivates the current study’s focus on Chinese EFL learners’ (CEFLLs) use of epistemic and effective modals in opinion-giving monologues—a task central to high-stakes speaking tests (e.g., IELTS, TOEFL) that demands nuanced stance expression [17].

## 2. Review of literature

### 2.1 Stance modals in written and spoken proses

Stance reflects a speaker’s epistemic and emotional attitudes toward the discussed issue, their confidence in the veracity, and source of information [1,6]. It enables the speaker to interact with addressees and manipulate the dialogic space for alternative viewpoints [2,3].

Most studies on modals’ role focus on epistemic modals, investigating their role in expressing authors’ commitment and establishing persona in written registers. Studies reveal the pervasiveness of hedging modals in research papers [2], serving to distinguish facts from opinions and present claims with appropriate caution and regard to peer views. While modal frequencies and choices may change [18], adept writers tend to hedge more than boost. In research articles, most epistemic markers (e.g., *might*, *may*, *could*, *possible(ly)*, *probably*, *likely*, *perhaps*) express uncertainty [9], functioning as a politeness strategy that acknowledges doubt and alternative propositions, thus respecting the readers’ negative face. The use of epistemic resources is influenced by social/cultural context [2]. Researchers examine student writings to emphasize the importance of evaluating assertions in acceptable and persuasive ways [15,19]. While inexperienced writers tend to boost rather than hedge [20,21], high-scored students used more hedges (e.g., *may*, *possibly*, *perhaps*) to establish cautious stance and critical distance and used boosters (e.g., *must*) with foregrounded evidence and reasoning to create authority and disciplinary engagement, reflecting the implicit values expected of students [22].

In contrast to the extensive literature on writing, studies on epistemic modals in speaking—particularly in academic contexts—are limited, despite their ubiquity in this register [6]. Biber [6] reveals the distribution of prediction, possibility, and necessity modals in college registers, and their interaction with communicative purposes. In 3-Minute Thesis (3MT) presentations, plausibility hedges (e.g., *could* and *may*) are prominent devices to signal claims established on plausible assumptions, thus mitigating assertion and acknowledging claims limitations while engaging the audience [23]. Comparison of stance markers across academic spoken and written genres represented by doctoral defenses and manuscript reviews reveals that booster modals, particularly with the first-person subject, are more prevalent in the spoken genre to create a robust authorial stance [8]. These studies show that modal choices interact with communicative purposes, yet further research is needed to explore the use of modals in various speaking contexts, especially in academic exchanges.

L2 learners’ modal use is also investigated, mainly focused on epistemic modals in writing, but the findings diverge in terms of the tones in their productions. Some researchers have observed that L2 learners generally follow established patterns. EFL learners from European L1 backgrounds displayed a similar tendency as the native speakers (NSs) regarding the use of epistemic stances [24]. Chinese student writers could tone down their language like L1-English experts in research papers [25], and they use more hedges than boosters in their dissertations to create a tentative persona [19,26–28]. However, other studies show that EFL learners tend to underuse [29] or overuse modals [30], creating a more tentative [31,32] or over-assertive tone [27,28]. Previous research has observed that CEFLLs tend to overuse modals like *can*, *could*, and *would* [33], while other studies have found that they underuse hedging epistemic modals [26], often marking unwarranted assertions and being suspicious of impoliteness with their particular favor for modals of high value and underuse of low-value ones [34]. While some studies find that L1 background has little effect on modal choices, with a similar preference observed among Turkish and Chinese learners [35], many researchers underscore the impact of L1 on EFL learners’ modals use in L2, including form-meaning relationships, rhetorical norms, and knowledge construction [12,33,36–38]. These findings suggest that L2 learners struggle with stance-taking. Nonetheless, the varying results highlight the need for further research to understand their use of epistemic modals for stance expression.

Additionally, previous literature primarily focuses on epistemic modals in writing, largely overlooking deontic modals and spoken discourse. Despite their relatively low frequency [39], deontic modals are essential for academic discourse and pose challenges for L2 learners. Researchers find that, possibly under L1 influence [40], EFL learners often overuse deontic modals to persuade or influence their readers [13,31,41,42] by frequently discussing personal or group obligations [43] and suggestions, which may result in weakened argumentation due to subjectivity [44]. This contradicts the typical practice of expert authors, who use deontic modals sparingly to avoid face-threatening [39,45]. However, frequency alone is insufficient to make the story clear, and the use of deontic modals needs examination, as studies suggest that their objective use predominates in essays [45].

Another overlooked issue is L2 learners’ use of modals in spoken discourse, which is abundant in modal stances but challenging for L2 learners, with limited and inconsistent literature in this field. Studies [17,46] show the prevalence of epistemic adverbial and lexical verbs in L2 speaking tests. However, “spoken interaction is especially challenging for L2 learners,” requiring real-time decision-making and response formulation ([47], p. 35). Consequently, they struggle to convey stances using appropriate form-function mappings [48]. Kärkkäinen [49] indicates that even the most competent Finnish L2 learners use fewer modals than NSs, although they frequently employ hedging epistemic modals for politeness. In opinion-giving interviews, Kizu et al. [47] find that epistemic modals are difficult for Japanese L2 learners with overseas study experience to master, unlike lexical verbs and adverbs. While Zhang and He [50] find that Chinese L2 learners used *must* seven times as often as NSs, Hu [51] indicates that their use of deontic modals is similar to NSs, with *must* being less common in their speech. These inconsistencies underscore the need for further investigation into the frequency and usage of modals.

A key distinction between written and spoken stance lies in interactivity and time pressure. In academic prose, epistemic modals (e.g., *may*, *could*) primarily serve to hedge claims and acknowledge alternative viewpoints [2]. In contrast, oral commentaries demand rapid integration of content planning and delivery, where modals function not only to express certainty or doubt but also to engage listeners persuasively [11]. For instance, in political speeches, speakers frequently deploy *must* to assert moral obligations (*We must act now*)—a strategy that aligns with the “effective stance” aimed at influencing reality [52]. Such findings underscore the need to analyze L2 learners’ modal usage beyond written norms, particularly under the cognitive constraints of impromptu tasks.

### 2.2 Form-focused stance studies

Studies of stance expression in academic discourse typically adopt two complementary approaches: framework-driven analyses and form-focused investigations. The former examines how stance categories (e.g., epistemicity, evaluation, engagement) are distributed across theoretical models such as Biber’s stance triad [1], Hyland’s metadiscourse framework [2], or Martin and White’s appraisal system [3]. These studies often focus on broad functional patterns, such as the prevalence of hedges (*may*, *slightly*) in mitigating claims or boosters (*must*, *clearly*) in asserting authority across disciplines [2,22], the preference for boosters and hedges under the influence of knowledge construction and rhetoric [38,53] and communication purposes [8,54].

Form-focused investigations target specific linguistic devices to uncover their stance-marking potential. Substantial research shows that nouns (e.g., *assumption*, *claim*) are interactive, convey evaluations, and reflect disciplinary and social changes [55–59]. Adverbials (e.g., *obviously*, *arguably*) signal epistemic certainty or evidential sourcing [60–62]. Pronouns (e.g., *we*, *you*) construct authorial presence and audience alignment, influenced by L1 writing conventions and perceptions of objectivity [63,64].

While these studies illuminate the role of nouns, adverbials, and pronouns in stance construction, modal verbs—particularly those expressing obligation (*must*, *should*) and potentiality (*can*, *could*)—require more focused attention in oral academic contexts, as modals are central to negotiating epistemic authority (e.g., *This finding may suggest...*) and pragmatic force (e.g., *Policymakers must act...*) in real-time discourse [52]. The “impromptu commentary task” in oral examinations of IELTS, TOEFL, and TEM-8 (a national test for English majors in China) requires candidates to articulate structured opinions on social or cultural topics within three minutes of preparation. Unlike academic writing, which prioritizes detached analysis and textual cohesion [2], this task simulates real-world scenarios where speakers must dynamically negotiate stance through linguistic resources such as modals, evidentials, and evaluative lexis ([1], pp. 966–972).

### 2.3 Modals in stance frameworks

Existing frameworks for stance analysis vary in their treatment of modal verbs. Biber et al. [1] categorize stance into three domains: epistemic (certainty/doubt), attitudinal (evaluative judgments), and style (interactional positioning). While this model acknowledges deontic modals (*must*, *should*) under “attitudinal stance” ([1], p. 975), it does not explicitly address their role in negotiating speaker accountability, which is common for oral discourse where directives (e.g., *we should*...) often serve persuasive goals. Hyland’s model [2], while valuable for written academic metadiscourse, treats persuasion as secondary to informational goals (e.g., hedging to avoid overstatement) and takes deontic modals as directives imposing obligations. However, in oral context, the speakers need to prioritize persuasion and often takes directives as means of invoking shared values.

Hidalgo-Downing’s [10] epistemic and effective stance framework addresses this limitation by distinguishing between: speaker’s assessment of propositional validity (epistemic stance, e.g., *pollution* ***may*** *be better controlled*) and speaker’s intent to influence events or listeners’ actions (effective stance, e.g., *we* ***should*** *reduce emissions*).

Although originally applied to political discourse [52], this framework proves adaptable to oral commentary tasks for two reasons: First, it highlights persuasive intention. Both political speeches and TEM-8 commentaries require candidates to legitimize viewpoints through modalized appeals (e.g., epistemic *might* to mitigate assertions vs. effective *must* to signal urgency). Second, it considers accountability. By classifying deontic modals as “effective”, the framework captures how speakers navigate responsibility—either foregrounding personal conviction (*I believe*...) or deflecting it through generalized claims (*People should*...) [11].

The current study utilizes Hidalgo-Downing’s [10] stance framework to investigate the role of epistemic and effective modals, as shown in Table 1.

**Table 1:** Hidalgo-Downing’s [10] framework of stance modals.

Modals for Epistemic stance
**Epistemic modals**: *may*, *might*, *could*, *will*, *would*, etc., which express a speaker’s assessment of the possibility, probability or certainty of a proposition or degree of commitment towards a proposition.
**Modals for Effective stance**
**Deontic modals**: *must*, *should*, *have to*, etc., which express obligation and necessity
**Potentiality modals**: *can* and *could*, which express participant internal or participant external possibility, ability or capacity

As mentioned above, previous studies on L2 modal usage predominantly focus on written texts, and few examine how learners negotiate epistemic and effective stance under the dual pressures of impromptu preparation and persuasive goals. By applying Hidalgo-Downing’s framework to TEM-8 oral commentaries, this study addresses two questions:

1. How do CEFLLs express epistemic stance with modals in terms of frequency, tones, and linguistic choices?
2. How do CEFLLs express effective stance with modals in terms of frequency and linguistic choices?

## 3. Dataset and methods

### 3.1 Corpus

The dataset for this study was drawn from two primary sources: (1) the SWECCL 2.0 [65], a set of speaking corpora of CEFLLs, and a self-compiled corpus of American college students [66], selected for their methodological complementarity despite temporal limitations.

The two corpora were accessed between September 2022 and December 2023 for research purposes. All corpora used in this study were anonymized prior to analysis, and no identifiable personal data were accessed during or after data collection.

SWECCL 2.0 remains one of the few publicly accessible Chinese learner corpora specifically designed for high-stakes oral proficiency assessments (e.g., TEM-8 Oral test), providing standardized monologic speech samples across argumentative topics. Its task alignment makes it indispensable for cross-group comparisons of stance markers in formal speaking contexts.

Qi and Ting’s [66] corpus, though dated, complements SWECCL 2.0 by offering native speaker (NS) data with comparable task constraints: both corpora feature 3-minute prepared commentaries on socially relevant or college-related topics, ensuring parity in discourse structure and communicative demands. While newer NS corpora exist, this dataset’s focus on commentary monologues—a genre directly mirroring the learner task—provides unmatched contextual consistency.

For the learner data, 150 transcribed speech texts were selected from a sub-corpus of SWECCL 2.0, especially from Task 3 of TEM 8-Oral (a computer-mediated, human-rated, and standardized national-level English test for fourth-year English majors in China) conducted between Years 2003–2007. This task provides printed prompts and were allotted a 4-minute preparation time before delivering a 3-minute commentary speech, or an opinion-giving monologue. The topics spanned social life, politics, economy, college life, and technology, with a full list provided in Appendix A in S1 Appendix.

To minimize the potential influence of learners’ proficiency levels on stance marker usage, a stratified sampling approach was adopted: an equal number of speech samples were drawn from three pre-classified proficiency groups (Excellent, Good, and Pass) within SWECCL 2.0. This design ensured that the analysis reflected general patterns of stance marker usage across learners rather than proficiency-specific tendencies.

Data processing involved removing filler words (e.g., *er* and *uhm*), stutters, repetitions, and self-correction, as well as correcting minor grammatical errors impacting stance coding. Unfinished sentences were also excluded. The edits were justified by their negligible impact on semantic content and stance analysis, aligning with prior methodology [67]. The final dataset comprised 14971, 13323, and 13213 words per proficiency level, totaling 41507.

The NS corpus (partially described in [66]) includes recorded talks of 40 students at Davidson College, USA, who delivered a 3-minute monologues on assigned topics after 3 minutes of preparation. The topics are listed in Appendix B in S1 Appendix, with a total production of 14802 words.

Crucially, both corpora share comparable task designs: timed monologues (3-minute speech after 3–4 minutes of preparation) on general-interest topics, recorded in controlled environments (e.g., standardized exam settings for learners vs. classroom recordings for NSs). These similarities ensure the validity of cross-croup comparisons in stance expression patterns. Furthermore, the topics in both corpora—either related to college life or of general interest—are inherently suitable for stance analysis, as they “elicit the linguistic expression of opinions, beliefs, feelings and personal experiences as well as an overall argumentative discourse” ([68], p. 1186).

Although the specific wording of prompts varied across corpora, all topics were designed to elicit argumentative discourse requiring speakers to articulate and defend personal viewpoints. Learner topics such as “China’s Employment Market Challenged by More Graduates” and “Pets or not?” and NS topics such as “Do you think it is appropriate for college students to rent apartments outside campus?” share core functional characteristics: they present a debatable issue, invite the speaker to take a position, and require justification of that position through reasoning and evidence. As Baumgarten and House [68] note, such topics are inherently suitable for stance analysis precisely because they encourage speakers to draw on linguistic resources for expressing opinions, beliefs, and evaluations. Thus, while prompt wording may differ in surface form, the underlying rhetorical demands are comparable across corpora.

### 3.2 Coding procedures

The coding of the data consisted of the following steps

**Step One**: Establish the tentative framework. The first author read 18 sample texts—three from NSs’ texts (one from each topic), and 15 from learners (one from each proficiency level of each topic). A tentative set of coding principles was established, and a list of excluded stance markers were compiled based on their occurrence in topic instructions or relevance to the topic.

**Step Two**: Invite experienced EFL teachers to annotate the samples

We invited two colleagues, both EFL teachers with over 25 years of experience, to code four texts from the Chinese learners’ sub-corpus that covered all target stance categories. They were provided with a theoretical framework, definitions, coding examples, task instructions, topic prompts, and a list of excluded markers. After independent coding, the intercoder reliability was about 89%, exceeding the “average agreement rate of approximately 80%” ([37], p. 2800). Following discussion and modifications, the inter-rater agreement increased to 93.5%. This rate was lower than Hu and Cao [37] (95.5% for hedges and 96.2 for boosters) and Jiang and Ma [69] (96%) but higher than 92.4% in [70]. For ambiguous items, assistance from colleagues and linguists was sought until a consensus was reached. A final framework was established based on the coding results and discussions.

**Step Three**: Annotate and re-annotate the data. Using the established framework, the first author coded the transcripts of all 150 learners and 40 NSs.

### 3.3 Coding criteria

The coding criteria focus on differentiating epistemic and effective meanings of some polysemic modals and defining boosting or hedging tones.

Epistemic meanings concern possibility, reflecting speakers’ attitudes toward the likelihood of a proposition ([71], p. 21; [72], p. 177). Following previous research [71,73], epistemic modals can be understood as expressing the speaker’s commitment to the likelihood of a proposition, ranging from possibility (*may*, *might*) through probability (*would*, *should*) to certainty (*must*, *will*). This continuum is reflected in the coding criteria summarized in Table 3, where each modal is assigned a specific value (e.g., *may* as hedging, *will* as boosting). As Palmer ([73], pp. 50–53) notes, *must* in its epistemic sense conveys a stronger inference, paraphraseable as “The only possible conclusion is that...” Common possibility modal verbs, adjectives, and adverbs include *will*, *would*, *may*, *might*, *could*, *maybe*, *perhaps*, and *unnecessary(ily)* (collectively referred to as modals in the study). Notably, *will, must*, *should,* and *can/could* may have different meanings depending on the context.

**Table 3:** Criteria for the tones of epistemic modals.

Markers	Tones	References
*must*	boosting	[20]
*will*	boosting	[15,20,74]
*should*	boosting	[20,74]
*probably*	boosting	[72]
*may, might, would*	hedging	[2,20]
*possibly, maybe*	hedging	[15]

#### Will.

This study distinguishes between its epistemic and dynamic meanings*,* based on whether the subject is predicating (epistemic) or declaring intention (effective). Epistemic *will*, the basic meaning ([1], pp. 495–596; [71], p. 126; [73], p. 135), refers to reasonable expectation and can be paraphrased as “A reasonable inference is that…” ([73], p. 57). Futurity *will* conveys epistemicity, reflecting “limitations to the speaker’s knowledge” ([72], p. 190) and predicting that a proposition will be valid in the future ([1], p. 979), as is shown in **Example 1**. Occasionally, *will* acts as an intentionality marker (e.g., **Example 2**), often with a first-person subject ([71], p. 193), under the assumption that “the subject-referent is in control” ([72], p. 193).

**Example 1** *Every single man* ***will*** *enjoy the benefit brought by the scientific improvement.* L1_03-020-25

**Example 2** I ***will*** *first bring into consideration of the safety of people of my city.* L1_06-004-19

#### *Must* and *should.*

*Must* and *should* are similar in that they often have a deontic sense but could convey epistemicity. The differentiation of epistemic and effective senses applies to both modals, and this section will take *must* as an example.

For *must,* epistemicity is a minor meaning ([71], p. 34), representing personal confident judgment (e.g., **Example 3**) and indicating “This is the only explanation I can think of” ([72], p. 181). By contrast, when *must* expresses obligation (e.g., **Example 4**), it reflects self-incitement, polite exhortation, advice, and request [71].

**Example 3** *Yes, as their... relatives, …****must*** *feel sad. L3_03-020-02*

**Example 4** *And this is the reason why we* ***must*** *give our most thank to all this[these] great martyrs and great scientists who contribute much to our happy life, improve the living conditions. L1_03-020-2*

#### *Can* and *could.*

When *can*/*could* conveys assessment of commitment to a proposition (usually with an inanimate agent), they serve as epistemic markers. Conversely, when *can*/*could* expresses internal or external possibility (neutral possibility) and ability, they function as effective markers for potentiality.

We distinguished ability and neutral possibility within effective *can/could*. First, the structure “*can* + sensation/private verb” (e.g., *see*, *understand*, *remember*, *think*, *afford*, *stand*, and *bear*) indicates ability ([71], p. 87). Importantly, *can* expresses ability when the realization of the predicate solely depends on the subject’s capability and willingness [75]. If the external conditions also play a role, *can* is interpreted as neutral possibility, though this differentiation is somewhat subjective.

In **Example 5**, the subject (a better education background) does not decisively influence outcomes (a better job/salary, a better workplace), which also depends on factors like employer recognition; thus, *can* indicates neutral possibility. In **Example 6**, *they* (pets) lack the cognitive power of humans, so *can(not)* indicates ability. In **Example 7**, although job acquisition isn’t solely decided by students, the speaker emphasizes with strong commitment that competent job-hunters possessed the ability to find jobs, and therefore *can* here is taken as ability.

**Example 5** *Getting higher education means you* ***can*** *get a better job, you* ***can*** *get better pay, and you* ***can*** *work in a better place. L1_ 04-005-29*

**Example 6** *They [referring to pets. Note is ours.]...****cannot*** *think just like human. L2_07-001-20*

**Example 7** *I am sure they [referring to graduates with good abilities] are capable they* ***can*** *find jobs. L2_04-005-14*

Additionally, we identified two other meanings of *can*: suggestion and permission, which were integrated into the framework due to their role in influencing reality. To control for topic-induced bias, all instances of suggestion *can* from the 2005 “Suggestions for the 2008 Beijing Olympics” prompt were excluded from the analysis. The suggestion *can* tokens reported in this study thus represent uses from other topics (e.g., the 2007 pet ownership prompt, the 2004 employment market prompt), where the task did not explicitly solicit suggestions. This exclusion ensures that the observed pattern of suggestion can reflects learners’ interlanguage tendencies rather than a purely task-induced artifact.

The coding criteria for modal verbs are summarized in Table 2.

**Table 2:** Coding criteria for epistemic and effective modals.

Markers	Categories	Criteria
*will*	Epistemic modality	prediction of possibility at present or in future; subject not in control of the realization
	Effective intentionality	expressing volition; subject in control of the realization
*must* *should*	Epistemic modality	judgment of possibility, confident inference
	Effective obligation	imposing obligation or granting permission,
*can*	Epistemic modality	“It is possible that…”; commenting on the possibility of the claim rather than the sentence subject
	Effective potentiality	“Someone has the ability to...”; subject in control of the realization; followed by sensation verbs and private verbs
		“It is possible for...”; subject not in control of the realization
		offering suggestion
		indicating allowing

To enhance the rigor of our categorization, we established criteria for distinguishing between “neutral possibility” and “suggestion” uses of *can*. Following previous research [75,76], we operationalized these categories as follows:

Neutral possibility *can*: The modal indicates that a state of affairs is possible given general circumstances, without explicit speaker endorsement or recommendation. Typically, the subject is inanimate or generic, and the utterance presents a factual possibility rather than a proposed course of action (e.g., “*Getting higher education means you can get a better job*”).

Suggestion *can*: The modal occurs with a first-person plural subject (*we*), followed by an action verb that proposes a collective course of action. The utterance functions as a recommendation or polite directive, often in the context of problem-solving (e.g., “*First of all, we can give ID card for every pet*”). Crucially, suggestion *can* implies speaker endorsement of the proposed action, whereas neutral possibility *can* merely states what is possible.

Obligation markers analyzed in this study include *should*, *must*, *have to*, *need to*, and other expressions of necessity or duty.

In addition to the meanings of modals, the hedging/boosting tones of epistemic modals were differentiated. As defined above, our analysis adopts a broad definition of “modals” that includes modal verbs, adjectives, and adverbs, all of which contribute to epistemic stance expression. The coding of these tones is based on previous studies, as shown in Table 3. As Hyland and Milton [15] note, categorizing modal certainty can be inevitably uncertain, so the results should be interpreted with caution.

### 3.4 Calculation and comparison of frequency and qualitative analysis

Due to the larger size of the learners’ sub-corpus (41,507 words) compared to the NSs’ (14,802 words), normalized frequencies were calculated and compared. The Chi-square test in SPSS was applied to determine significant differences between frequencies, with significance set at p < 0.05.

Additionally, qualitative analysis of concordance lines was conducted using AntConc to examine sentence subjects and verb types for selected modals (e.g., *can*, *should*, *must*) where contextual information was necessary for interpreting functional distinctions.

### 3.5 Ethics Statement

This study is based entirely on pre-existing, licensed corpus data. It does not involve any human participants, personally identifiable information, or intervention. Therefore, ethical approval and informed consent were not required.

## 4. Results

### 4.1 RQ 1: How do CEFLLs use modals for epistemic stance?

CEFLLs exhibited a marked preference for boosting over hedging in epistemic stance expression, contrasting with NSs. They used fewer epistemic modals than NSs (104 vs. 157; *χ*^2^ = 10.762^a^, *p* = 0.001, Table 4). They preferred boosting epistemic modals (73 vs. 31; *χ*^2^ = 16.962^a^, *p* *<* *0.001*), while NSs favored hedging (39 vs. 118; *χ*^*2*^ = 39.752^a^, *p < 0.001*). Meanwhile, compared with NSs, learners used more boosting modals (73 vs. 39; *χ*^2^ = 10.321^a^, *p* = 0.001) and fewer hedging (31 vs.118; *χ*^2^ = 50.799^a^, *p* *<* 0.001). This suggests learners’ possible reliance on high-certainty markers to compensate for limited hedging strategies.

**Table 4:** Frequencies of epistemic modals (per 10,000 words).

	Markers	Learners	NSs
**Boosting modals**	*will*	70	28
	necessary/necessarily*	2	10
	*must (possibility)*	1	1
	**Sub-Total**	**73**	**39**
**Hedging modals**	*may*	12	4
	*maybe*	9	14
	*would*	5	60
	*might*	1	11
	*could*	1	5
	*probably*	0	18
	*Others*	3	6
	**Sub-Total**	**31**	**118**
	**Total**	**104**	**157**

*Note: 1. Following the definition in Section 3.3, the term “modals” in this table encompasses modal verbs, adjectives, and adverbs. 2. The category “**necessary/necessarily**” combines both the adjective **necessary** and the adverb **necessarily**, as both function as boosting markers. CEFLLs used only **necessary** (2/10,000 words); NSs used 4 **necessary** and 6 **necessarily** per 10,000 words.

Both groups used similar boosting markers (*will*, *necessary(ily)*, *must*), but learners favored *will* (69 vs 28; *χ*^2^ = 17.33^a^, *p <* 0.001, Table 4) and underused *necessary(ily)* (2 vs 10; *χ*^2^ = 5.333^a^, *p* = 0.021). For hedging devices, CEFLLs primarily used *may, maybe,* and *would*. They used fewer *would* (5 vs. NS’s 60; *χ*^2^ = 46.538^a^, *p <* 0.001, Table 4) and *might* (1 vs. NS’s 11; *χ*^2^ = 8.333^a^, *p* = 0.004) but more *may* (12 vs. NS’s 4; *χ*^2^ = 4^a^, *p* = 0.046). Notably, *probably*—a common NS hedge—was absent in learner discourse. No significant difference was found in the frequencies of *could* (1 vs. 5; *χ*^2^ = 2.667^a^, *p* = 0.102) and *maybe* (9 vs. 14; *χ*^2^ = 1.087^a^, *p = 0.297*).

The overuse of boosting modals (*will*, *must*) and limited hedging repertoire (*may*, *maybe*) indicate that CEFLLs prioritize assertive stance-taking, possibly due to L1 transfer or task pressure in impromptu speaking. This contrasts with expert writers’ preference for hedging to acknowledge dialogic space [18].

### 4.2 RQ 2: How do CEFLLs use modals for effective stance?

CEFLLs used more effective modals than NSs (174 vs. 105, *χ*^2^ = 17.065^a^, *p <* 0.001, Table 5). First, they employed more obligation modals (113 vs. 59, *χ*^2^ = 16.953^a^, *p <* 0.001). This aligns with studies on L2 learners’ tendency to impose obligations for persuasive goals (13). Second, while both groups used *can* for ability (10 vs. 9, *χ*^2^ = 0.053^a^, *p* = 0.819), neutral possibility (39 vs. 34, *χ*^2^ = 0.342^a^, *p* = 0. 558), and permission (4 vs. 3, *χ*^2^ = 0.143^a^, *p* = 0.705) with non-significant statistical difference, learners uniquely deployed *can* for suggestions (8 vs. NSs’ 0).

**Table 5:** Total frequencies of effective markers (per 10,000 words).

Types	Sub-type	Learners	NSs
Obligation	/	113	59
Potentiality	Ability	10	9
	Neutral possibility	39	34
	Permission	4	3
	Suggestion	8	0
	**Sub-total**	**61**	**46**
**TOTAL**	**/**	**174**	**105**

Further analysis showed some contextual nuances in use of *can*. To explore these patterns, we distinguished between circumstantial and non-circumstantial occurrences of *can*. Circumstantial can refers to instances where the possibility expressed by *can* is contingent on external conditions, often signaled by conditional clauses (*if*-clause), prepositional phrases (*in this way*, *through*), temporal adverbials (*sometimes*), or other expressions that indicate conditions for possibility. In contrast, non-circumstantial *can* presents possibility as general or inherent, without such contextual constraints.

First, CEFLLs preferred conditional over non-conditional uses (24 vs. 12; *χ*^2^ = 4.000^a^, *p* = 0.046), opposite to NSs (26 vs. 8; *χ*^2^ = 9.529^a^, *p* = 0.002, Table 6). Moreover, they used more conditional (24 vs. NSs’ 8; *χ*^2^ = 8^a^, *p* = 0.005) and fewer non- conditional uses (12 vs. NSs’ 26; *χ*^2^ = 5.518^a^, *p* = 0.023). This suggests learners’ focus on external conditions to justify claims, contrasting with NSs’ reliance on internal reasoning.

**Table 6:** Frequencies of various uses of potentiality modals (per 10,000 words).

Sub-Types	Uses	Learners	NSs
Ability	+ action verbs	5	6
	+ private verbs	5	3
	**Sub-total**	**10**	**9**
Neutral possibility	circumstantial	24	8
	non-circumstantial	12	26
	emphatic	3	0
	**Sub-total**	**39**	**34**

Learners introduced an “emphatic” use of *can* (3 tokens) through rhetorical questions, a strategy absent in NS data. This highlights learners’ adaptive use of modals for persuasive emphasis under time constraints.

Table 7 presents the frequencies of common effective markers, including the obligation markers *should*, *must*, *have to*, and *need to*. Regarding effective modal markers, CEFLLs favored *should* for obligation, while NSs preferred *have to*. Leaners used more *should* (84 vs. 17, *χ*^2^ = 44.446^a^, *p <* 0.001, Table 7) and *must* (13 vs. 1, *χ*^2^ = 8.067^a^, *p* = 0.005) but fewer *have to* (10 vs. 31, *χ*^2^ = 10.756^a^, *p* = 0.001) than NSs. *Need to* did not significantly differ between groups (4 vs. 10, *χ*^2^ = 2.571^a^, *p* = 0.109). *Can* was the sole choice for potentiality, while *could* was not identified.

**Table 7:** Frequencies of common effective markers (per 10000 words).

Types	Markers	Learners	NSs
**Obligation/** **necessity**	*should*	84	17
	*must*	13	1
	*have to*	10	31
	*need to*	4	10
	*others*	2	0
**Potentiality**	*can*	61	46

## 5. Discussion

### 5.1 Epistemic modal underuse

Despite the prevalence of epistemic and effective modals, this study revealed that CEFLLs faced challenges in using epistemic modals, aligning with prior findings on NNSs’ difficulty in acquiring epistemic modality [9,77,78]. One reason may stem from the context-dependent meanings and functions of epistemic modals in English, making it hard to establish a one-to-one form-function relation [15] and complicating acquisition for EFL learners [35,73,79]. Meanwhile, epistemic modals implicitly shape the speaker-audience relationship, positioning the audience as passive or allowing for negotiation. This subtle intention requires understanding of cultural and rhetorical norms in the target language, posing further challenges for L2 learners [80].

Another possible reason is that CEFLLs appear to lack adequate pedagogical support in epistemic modality. English textbooks have been found with limited explicit instruction on epistemic modality [81,82] and alternative devices [83–85], which also occur in EFL textbooks from other L1 background [24,86]. Also, EFL teachers claimed to fail to provide alternatives to categorical assertion [15] or training tasks [86]. Consequently, learners struggle to express opinions with appropriate commitment, often sounding overassertive.

Furthermore, epistemic modality is closely tied to culturally shaped expectations of epistemic responsibility, the degree to which speakers or writers are expected to commit themselves to the truth value of a proposition. In western academic traditions, speakers/writers are often expected to demonstrate awareness of the limits of their knowledge, as a form of intellectual modesty and accountability [37]. This encourages the use of hedging modals and adverbs to qualify claims and mitigate potential overgeneralizations. In contrast, Chinese academic and rhetorical traditions have historically placed less emphasis on individual epistemic responsibility and more on aligning with authoritative knowledge or consensus [12,82]. As a result, CEFLLs may be less inclined to soften their claims with epistemic markers, often opting to present knowledge more categorically. This underlying epistemological stance can contribute to the underuse of epistemic modals in CEFLLs’ speech, as learners may not perceive such mitigation as necessary or even appropriate.

However, we acknowledge that this cultural explanation is not the only possible interpretation of the observed patterns. While we have suggested that learners’ reliance on high-certainty markers may reflect compensation for limited hedging strategies, we acknowledge that this interpretation is inferential and that multiple explanations remain possible. First, as previous research has noted, Chinese rhetorical traditions may favor directness and assertiveness, leading learners to adopt a genuinely assertive stance rather than a compensatory one [82,87,88]. Second, the nature of the opinion-giving task itself—which requires speakers to articulate and defend positions—may invite stronger epistemic stances than more interactive or negotiated discourse contexts [17,23]. Third, lower-proficiency learners may simply default to high-frequency, early-acquired modals such as *will* under task pressure, regardless of hedging intent [89]. These possibilities are not mutually exclusive, and future research employing introspective methods (e.g., stimulated recall) could help disentangle learners’ underlying intentions.

Importantly, the patterns observed in this study—particularly learners’ preference for boosting modals (*will*, *must*) and their overuse of deontic should—are not isolated to the specific dataset examined. Similar tendencies have been reported in more recent studies on Chinese EFL learners’ modal use [33,78], suggesting that these features may reflect relatively stable characteristics of Chinese learners’ interlanguage rather than transient phenomena tied to a particular time period. This interpretation aligns with research on second language fossilization, which indicates that certain grammatical and pragmatic features—especially in the domain of modality—can become entrenched in learners’ interlanguage systems and persist despite changes in instructional approaches or input exposure [90,91]. Thus, while the data in this study are drawn from the 2003–2007 period, the patterns identified may offer insights into enduring challenges in Chinese EFL learners’ spoken stance-taking.

### 5.2 Challenges of hedging epistemic modals

The higher frequency of *will* and the infrequent *would* in CEFLLs’ speech (Table 4) supports the conclusion that learners prefer boosting strategies, aligning with previous findings on CEFLLs’ preference for stronger modals and an authoritative tone [42,70,78,92], a tendency often found among inexperienced English speakers [21]. We propose a multifactorial explanation for CEFLLs’ preference.

The first reason may stem from the transfer of Chinese rhetorical tradition, where writers are granted authority to use amplification to enhance conviction [82], preferring high-value modals to assert stance [87] and taking weak modals as weakening claims [88]. CEFLLs may have adopted an assertive tone with boosting modals to increase convincingness, without awareness that debates contribute to knowledge construction in Western traditions [93] and writers are expected to avoid overstatements and mitigate their claims [94].

Second, the L1-L2 form-function asymmetry may lead to CEFLLs’ insensitivity to certainty scales in English due to the absence of verb tenses in their L1. In Chinese, weak modality is expressed through verbs and adverbs rather than verb tense changes. The Chinese equivalent of *would* and *will* is the same, *jiang* (将) [70]. As shown in **Examples 8–9**, learners conflate *will* and *would* due to Chinese *jiang* (将) covering both meanings, leading to pragmatic infelicities [70] as well as tonic inconsistency, a common issue among EFL learners [15,31].

**Example 8** *If I were the mayor, I* ***would*** *allow my citizens to organize the celebration. That is, I* ***will****... arrange a specific time and several areas during the Spring Festival for people to gather to celebrate it. L1_06-004-22*

**Example 9** *We* ***are sure*** *in the future we* ***would*** *have more excellent... asterers [astronauts, the note is mine] and we* ***will*** *make closer to discovering of the mysteries of the universe. L2_03-020-20*

Third, task pressure may also result in the rise of *will*. Under timed conditions, learners may default to high-frequency, early-acquired modals like *will*. For CEFLLs, the predictive modal *jiang* (将) is more closely related to *will* than *would*, since *will* is acquired earlier and hard to be replaced due to form-meaning mapping [89].

In addition to these factors, learners’ infrequent use of *might* and *would* may also reflect avoidance strategies. As noted in previous research [47], L2 learners may avoid certain modal forms to minimize the risk of errors that could mark them as non-native speakers. Chinese EFL learners may be particularly unfamiliar with the hypothetical or tentative functions of *would* and *might*, as these nuanced uses receive limited attention in instructional materials [81–83] and are less salient in input compared to high-frequency modals like *will* and *can* [77,78]. Under time pressure, learners may therefore default to more familiar forms rather than risk using less confidently acquired modals. This avoidance, combined with the form-meaning mapping challenges [89], may contribute to the underuse of these hedging devices observed in this study. Future research employing introspective methods could help determine whether learners actively avoid these forms due to uncertainty or simply default to more accessible alternatives under task pressure.

To mitigate overgeneralization, we contextualize this finding within broader linguistic patterns. While boosting is prevalent, learners occasionally used *may* and *maybe* (12 and 9 tokens per 10k words, Table 4) to soften claims, indicating emergent hedging awareness and also limited resources for possibility [33]. Learners rarely use epistemic adverbs, like *necessarily*, *probably*, *possibly*, which not only modulate evaluative tone [8], but also serve as “fairly conventionalized indications of politeness behaviour” ([49], p. 206).

*Maybe*, the only likelihood adverb used by CEFLLs in this study may also prevent learners from acquiring similar adverbs. As the most common epistemic adverb in English ([1], p. 562; [95]), *maybe* is the first and the most frequently used epistemic modal for EFL learners [47] due to its simple pronunciation, clear meaning, and flexible position [77,78]. However, reliance on *maybe* might wane learners’ passion for picking other adverbs like *probably* and *perhaps*, a phenomenon known as blocking [96].

CEFLLs’ preference for boosting epistemic modals stands in contrast to the modal choices observed in learners from other cultural backgrounds. European learners, for example, have been found to show a similar degree of epistemic moderation as native speakers through their use of modal verbs [24]. German, French, and Swedish learners even used more possibility modals than NSs in some contexts [31]. These patterns differ from CEFLLs’ more assertive modal choices, suggesting a culturally shaped rhetorical stance that prioritizes conviction and clarity. Such cross-cultural variation highlights the importance of explicit instruction in hedging strategies, particularly for learners from rhetorical traditions that value strong, direct assertions.

While multiple factors—including L1 transfer [12,82], cultural rhetorical norms [87,88], pedagogical limitations [81–83], and task pressure [89]—contribute to CEFLLs’ modal use patterns, it is important to consider how these factors interact rather than operating in isolation. L1 transfer and cultural norms may predispose learners toward assertive stance-taking, while limited pedagogical exposure to hedging devices [81–83] fails to provide alternatives. Under task pressure [89], learners then default to the most accessible forms—often those reinforced by both L1 and instruction (e.g., *will*, *should*). Thus, these factors are mutually reinforcing: cultural predispositions are compounded by instructional gaps and exacerbated by performance conditions. Among these, pedagogical factors may be the most amenable to intervention, as they can be addressed through curriculum design and teacher training [15,86], suggesting that targeted instruction in hedging strategies could help learners expand their modal repertoire even if L1 influence persists.

### 5.3 Effective stance: cultural pragmatics of obligation modals

CEFLLs used more effective modals than NSs (174 vs. 105 tokens per 10k words, Table 5), particularly deontic modals (113 out of 174 tokens), which highlights necessity and legitimacy of actions [52] and an explicit persuasive or coercive tone [11]. Obligation modals were nearly twice as frequent in CEFLLs’ speech as in NSs’, supporting prior findings on learners’ overuse of obligation modals [13,19,40,44,50]. It is understandable that learners would default to familiar forms like *should* and *must* which they have acquired in early stage [40,44] under task pressure in speaking tests. Meanwhile, the high frequency of deontic modals is likely tied to learners’ L1 cultural values. Understanding of obligation is culturally bound [97], leading learners to transfer L1 norms to L2 [12] and use deontic modals in a way reflecting their own cultural values [98]. While obligation modals in English imply compulsion and can be pragmatically risky [21], CEFLLs often use obligation modals to show consideration for others and societal commitment [98] and to highlight responsibility and necessity [50] as Chinese culture prioritizes collective honor and benefits.

*Should*, the most common effective marker in the study, signals moral or social recommendations rather than absolute obligations. Aligning with previous studies [13,41,42,92], learners used *should* nearly five times as many as the NSs (84 vs. 17, Table 7), displaying attempts to influence audience in a direct and emphatic style [31] by resorting to social expectations [12].

A review of the distribution of *should* and *must* across the five learner topics (2003–2007) suggests that while the “Suggestions for the 2008 Beijing Olympics” prompt may have contributed to the high frequency of these modals—particularly *must*, which peaked in 2005—their frequent use was not confined to this topic alone. For instance, *should* appeared consistently across topics, with comparably high frequencies in the 2004 employment market prompt (70 tokens) and the 2006 firecrackers debate (96 tokens), the latter of which did not explicitly solicit suggestions but invited evaluative argumentation. This cross-topic consistency suggests that learners’ reliance on obligation modals reflects a more general tendency rather than a purely prompt-specific artifact, although the influence of topic cannot be entirely discounted. This interpretation aligns with previous research documenting Chinese EFL learners’ preference for deontic modals across various task types [13,41,42,92].

In **Example 10**, the repeated use of *should* reflects socio-centric obligation [98], where learners frame suggestions as communal duties (e.g., improving skills for collective competitiveness). This tendency aligns with findings on EFL learners from other collectivist cultures [99,100]. However, this pattern also highlights pedagogical fossilization. Textbooks also highlight *should* for suggestions [33]. For example, English textbooks for primary school translate *should* simply into *yinggai* (应该) that in Chinese aligns with group-oriented advice, without highlighting its implication of forceful suggestions or prohibitions ([71], p. 45) or providing its common hedges like *I believe/think* [97]. Such direct translation may oversimplify subtle distinctions in meaning, such as the difference between *should* and *could* when making tentative suggestions.

**Example 10** *We* ***should*** *have right attitude towards this tense this tough working situation... Also, we* ***should*** *evaluate our ability and choose the suitable job according to our interest and capability... and in our days, as most companies needs comprehensive people, so we* ***should*** *improve our ability in several aspects, such as English and computer, both* ***should*** *be reached at a high level. and except our... own major, we* ***should*** *learn... more things on different fields, such as we* ***should*** *have some knowledge of laws, of medicine, of computer and so on..*.*. I think the way to change our attitude, is that we* ***should*** *see clearly what kind of level ourselves is... L1_04-005-02*

Besides *should*, CEFLLs also frequently used *must*. Although *must* is considered “too strong for face-to-face contexts” ([6], p. 105), it is frequently used by EFL learners [13,31,44,50]. In **Example 11**, the learner repeatedly used *must* to emphasize strict requirements for pet owners, as deontic modals like *must* function to “impose obligation or necessity” [11]. While this suggests an authoritative stance, it is also possible that the learner defaulted to must as a familiar high-frequency modal due to limited lexical resources, a limitation inherent to corpus-based research as noted in Section 6. However, the overuse of *must* may stem from L1 transfer effects: in Chinese, the equivalent *bixu* (必须) often conveys communal responsibility rather than legal compulsion and can be used to offer suggestion and set obligation but with much weaker force [81], leading learners to misjudge the pragmatic force of *must* in English. Yet, such usage risks sounding overly forceful, as *must* in English implies non-negotiable mandates (e.g., legal obligations) rather than collective suggestions. Moreover, CEFLLs might overuse *must* to compensate for limited language proficiency, as amplification is a typical strategy for L2 learners facing linguistic challenge [82]. However, like *should*, this emphatic style may reduce persuasion, as what CEFLLs assume as axiomatic truths may differ in English-speaking cultures [12].

**Example 11** *First point is that you* ***must****...clean them because they are dirty. They can’t control themselves. They can’t clean themselves enough. And the next is you* ***must*** *to train them because if they bite others, you may be sued. And this is not as easy as you think. You …may get some publish according to the laws. So, raise the pet have many many disadvantages. So, I think we* ***must*** *control the numbers the people raise pets …and do the preparations before you…rise pets especially cats and dogs. L2_07-001-03*

CEFLLs not only used more deontic modal verbs but also tended to use them subjectively. About half of the concordance sentences with *should* and *must* featured the plural subject, *we*, as shown in **Examples 12** and **13**. *We should* reflects “what the speaker considers ‘right’” ([72], p.186), and *we must* conveys a stronger authority than an objective third-person usage ([71], p. 38), expecting compliance to some degree [8]. Asian EFL learners use *we must/should* to express “a strong mindset in regard to how one can be a contribution to one’s society” ([98], p. 33) and “self-discipline and responsibility” to society, along with the “pride” of contributing to it (p. 34).

**Example 12 *We should pay more attention to*** *this, and* ***we should begin from improve*** *[improving, note is mine] our people’s quality. L1_04-005-23*

**Example 13** *The second suggestion is that* ***we must improve*** *our environment. L1_05-012-15*

In contrast to CEFLLs, NSs used *should/must* with a third-person subject. The most frequent subject for *should* is *that* (e.g., **Example 14**), reflecting the speaker’s individual preference [13] rather than a collective belief. Only two occurrences (raw frequency) of *must* were identified in NSs’ speech, used with passive voice verbs (**Example 15**). The claim sounds objective, as “an agentless passive with an unspecified deontic source ha[s] no necessary connection with the speaker, where *must* merely expresses what is thought to be desirable” ([71], p. 38).

**Example 14** *The age of 21, 22, you know, 19, that you are really capable of making a commitment* ***that should last your entire life****.... Plus, I mean, marriage is a very very very important decision, something* ***that should be weighed with very very great care****. NS_S.C.19*

**Example 15** *I just think there’re good things [in using electronic dictionaries, the note is mine] in general, that* ***they must be verified*** *and* ***must be sited*** *[cited, the note is mine]* ***properly****. NS_S.E.21*

This divergence in the use of obligation modals between CEFLLs and NSs reveals deeper cross-cultural differences in how commitment and authority are expressed. In formal Chinese discourse, strong modal expressions (e.g., 要, 必须 [*have to, must*]) are commonly used to assert duties or collective values, reflecting hierarchical and speaker-centered communication norms. When translated into English, however, such expressions are often softened or omitted altogether, typically replaced by median-valued modals that better align with English preferences for negotiation, individual autonomy, and politeness [87]. CEFLLs’ reliance on modals like *must* and *should* may therefore reflect culturally rooted assumptions about persuasiveness that, in English contexts, can sometimes feel overly forceful. These findings underscore the importance of developing learners’ awareness of modal pragmatics in intercultural communication and EFL instruction.

While the high frequency of deontic modals in learner speech may be partly attributable to the nature of specific prompts (e.g., the “Suggestions for the 2008 Beijing Olympics” topic), cross-topic consistency suggests that additional factors are at play. Even in topics that did not explicitly solicit recommendations—such as the 2006 firecrackers debate and the 2007 pet ownership topic—learners frequently employed *must* and *should* to frame personal opinions as collective obligations (see Examples 10–11). This pattern aligns with previous research on Chinese EFL learners’ modal use [13,41,42,92] and may reflect culturally shaped rhetorical norms that prioritize social responsibility and collective action over individual hedging [98]. By contrast, NSs in comparable argumentative tasks (e.g., “Do you think it is appropriate for college students to get married?”) tended to employ more epistemic modals and impersonal constructions (see Examples 14–15), suggesting a preference for distancing strategies that mitigate personal accountability. Thus, while prompt effects cannot be entirely discounted, the cross-topic persistence of these patterns points to deeper interlanguage tendencies rooted in L1 rhetorical traditions and pedagogical practices.

### 5.4 Effective stance: *can* as indicator for mitigation and assertiveness

Previous studies on the frequency of *can* used by CEFLLs have yielded mixed results: some reported overuse [40,41,43,44], while others found no significant differences [13,92]. This study suggests that these discrepancies may arise from the senses of *can* investigated. Normalized frequencies of *can* were similar between groups in the senses of ability or permission, but learners used more instances of neutral possibility and suggestion. Meanwhile, they employed *can* with conditions or for suggestion to mitigate firmness, while using it emphatically for assertiveness.

First, CEFLLs used *can* to avoid being categorical, often with conditional clauses (e.g., *if*-clause, prepositions like *in this way, through, without*), hereafter briefly called “conditional *can*”. Conditions are considered a polite way to make suggestions [76], often used by EFL learners [100]. The high rate of conditional *can* also reflects the influence of the Chinese language, where speakers use conditions to present claims as objective and less open to criticism. When writing in English, CEFLLs often translate Chinese modals *hui* (会) and *neng* (能) as *can*. Research indicates L1 influences CEFLLs’ use of *can* with conditions [101,102]. Similarly, Japanese EFL learners often translate their potential markers into *can, be able to, possibly*, or *perhaps* to soften personal judgment [103].

Second, CEFLLs often used “suggestion *can*” to mark effective stance, a feature unique to their speech. The frequent phrase *we can* (e.g., **Example 16**) functions as a collective directive, reflecting the Chinese three-step argumentative structure (propose → analyze → solve), where, in the last step, they often use “*women keyi*” (*we can*) to show initiative and involvement and make suggestions more courteous and acceptable. Under the influence of L1, learners often discuss potential actions with *we can* [41] in their English argumentation due to an “overreliance on direct translation from Chinese” ([104], p. 6). The use of *we can* indicates an awareness of mitigation compared to proposing suggestions with *should* or *must*. The phrase makes suggestions tentative and negotiable, since it indicates an offer of possible options [100]. However, the overuse of *we can* reveals limited hedging strategies as noted in prior studies [41,100].

**Example 16** *First of all,* ***we can*** *give ID card for every pet… we* ***can*** *give these pets health test and we* ***can*** *make them very healthy and clean and no harm for human. So for this kind of pets, we* ***can*** *give them ID cards. L2_07-001-27*

One might question whether the “suggestion *can*” identified in this study is merely an artifact of the 2005 “Suggestions for the 2008 Beijing Olympics” prompt. However, two observations suggest otherwise. First, as noted in Section 3.3, all instances of suggestion can from the Olympics prompt were excluded from the analysis; the tokens reported here were drawn from other topics, including the 2004 employment market prompt and the 2007 pet ownership prompt, which did not explicitly solicit recommendations. Second, the frequent phrase *we can* (e.g., Example 16) reflects a broader discourse pattern in Chinese learners’ argumentation: the tendency to propose collective solutions in the problem-solving stage of their three-step rhetorical structure (propose → analyze → solve) [104]. This pattern, influenced by L1 rhetorical conventions, appears across topics rather than being confined to suggestion-specific prompts. Thus, while prompt effects cannot be entirely ruled out, the presence of suggestion *can* in non-suggestion topics suggests that it represents a genuine interlanguage feature rather than a purely task-induced artifact.

Third, CEFLLs sometimes use *can* to show strong emotions, a feature absent in the NSs’ speech. They employed *can* in rhetorical questions (e.g., **Examples 17**) or emphatic sentences (e.g., **Example 18**) to highlight their firm stance. Rhetorical questions indicate negation, are subjective, and convey strong emotions [105]. In this context, *can* amplifies subjectivity and emotion, reflecting the speaker’s firm positioning.

**Example 17** *Without life, how can we explore the universe, how can we develop the country and how can we try out [our] best to help the mankind? L2_ 03-020-04*

**Example 18** *Only in this way can they be compitive [competitive] in the market. L2_04-005-04*

The distinctive use of *can* by CEFLLs—both as a softener and a marker of assertive stance—reveals how learners navigate between languages and cultural expectations. Rather than conveying categorical certainty, which is typical in Chinese formal discourse as mentioned above, expressions like *we can* tend to offer options, invite cooperation, and help mitigate imposition, aligning more closely with English rhetorical norms that emphasize dialogue and flexibility. The frequent use of *conditional can* and *suggestion can* indicates an attempt to present arguments as reasoned and balanced, echoing strategies of politeness and reader engagement valued in English academic discourse. This tendency to tone down or reframe modal force resonates with translators’ practices of omitting high-value modal expressions and adding median-value ones in Chinese-English translation [87]. While this reflects an encouraging shift toward target-language norms, the learners’ limited repertoire of hedging devices also underscores the need for more comprehensive instruction in expressing stance effectively across cultures.

## 6. Conclusion

This study investigated the use of epistemic and effective modals in Chinese EFL learners’ (CEFLLs) argumentative speech, revealing distinct patterns compared to native speakers (NSs). Key findings demonstrate that CEFLLs employed fewer epistemic modals (e.g., *would*, *might*) but more effective modals (e.g., *should*, *must*), resulting in an authoritative and assertive tone. However, these patterns should be interpreted with caution given the potential influence of topic type, which was not systematically controlled in this study (see Limitations). Their overreliance on high-certainty markers (*will*, *must*), first-person subjects, and evaluative verbs reduced discourse objectivity, while mitigation strategies such as conditional *can* and suggestion *can* were applied. These patterns reflect the interplay of L1 transfer, pedagogical gaps, and task constraints, as discussed in the preceding sections.

The findings align with Hidalgo-Downing’s [10] epistemic-effective stance framework while expanding its applicability to EFL learners. Notably, the identification of “suggestion *can*” and “circumstantial *can*” in CEFLLs’ speech highlights categories absent in the original framework, suggesting the need for more nuanced modal classifications in learner language analysis. This contribution opens avenues for future research on how EFL learners negotiate stance in real-time oral tasks, particularly under cognitive pressures.

Pedagogical implications of this study are threefold. First, EFL instruction should prioritize raising learners’ awareness of stance gradation. CEFLLs’ tendency to adopt a firm tone through obligation modals (e.g., *must*, *should*) risks miscommunication with Western audiences, where over-assertiveness may weaken persuasion. Explicit teaching of hedging devices (e.g., *could*, *possibly*, *perhaps*) and contextualized practice in mitigating claims are essential. Second, curriculum designers should diversify modal instruction beyond form-focused approaches. For instance, contrasting L1 and L2 pragmatic norms (e.g., Chinese *bixu* vs. English *must*) could reduce fossilized errors. Finally, task-based activities simulating high-stakes speaking tests (e.g., TEM-8 monologues) should integrate training on modal flexibility, emphasizing how *can* and *would* can balance assertiveness and politeness.

This study has several limitations that warrant attention. First, due to the corpus-based nature of the research and the unavailability of original speakers, it was not possible to collect interview data. This limits the ability to explore learners’ underlying intentions or cognitive processes in their use of modal verbs. Future research could consider compiling smaller-scale spoken corpora that include self-reported data, such as stimulated recall or retrospective interviews, to provide richer insights into speaker intent.

Second, the learner data in this study were drawn from the SWECCL 2.0 corpus, specifically from TEM-8 tests conducted between 2003 and 2007. Although these data are approximately two decades old, SWECCL 2.0 remains one of the few publicly accessible Chinese learner corpora designed for high-stakes oral proficiency assessments, providing standardized monologic speech samples essential for cross-group comparisons of stance markers. Nevertheless, we acknowledge this temporal limitation and interpret our findings as reflecting the modal use of CEFLLs during that period. Importantly, research on second language fossilization suggests that certain interlanguage features—particularly in the domain of modality—may exhibit stabilized patterns that persist over time despite changes in instructional approaches or input exposure [90]. Future studies should verify whether the patterns observed here hold for more recent cohorts of Chinese EFL learners using updated corpora.

Third, while both corpora featured comparable task designs—timed monologues on opinion-eliciting topics in controlled settings—topic variation was not systematically controlled, and this remains a potential confounding factor. Although the topics were thematically similar (e.g., college life, public issues) and selected for their suitability in eliciting stance expressions [68], the specific wording of prompts may have influenced modal choices. For instance, the learner prompt “Suggestions for the 2008 Beijing Olympics” explicitly solicits recommendations, which may naturally elicit deontic modals such as *should* and *must*, while NS prompts framed as “Do you think it is appropriate...” may favor epistemic evaluations. Previous research has documented the significant impact of essay topics on modal verb usage in both L1 and L2 writing [13]. To mitigate this potential bias, we excluded data from the Olympics prompt when analyzing *suggestion can*, as this topic might have disproportionately influenced that specific usage. However, we acknowledge that this topic may have also contributed to the higher frequency of other deontic modals, particularly *should* and *must*, in the learner corpus. While a review of concordance lines across other learner topics (e.g., “Pets or not?”, “Should firecrackers be allowed during Spring Festival?”) suggests that the preference for obligation modals was not confined to the Olympics prompt alone, we cannot fully rule out the influence of topic effects. Still, we acknowledge that topic variation remains a potential confounding factor, and the lack of systematic control limits the generalizability of our findings regarding the influence of topic type (e.g., public issues vs. personal experiences) on modal choices. Future research would benefit from stricter topic control or within-topic comparisons across learner and native speaker groups to isolate the effects of prompt wording from genuine interlanguage features.

Fourth, although the original spoken data included audio recordings, this study focused solely on the analysis of transcribed speech. As a result, prosodic features such as intonation, stress, and pitch—which play a significant role in shaping stance—were not examined. This methodological decision was made to maintain consistency and manageability within the scope of the current study. Future research could build on these findings by incorporating acoustic analyses to offer a more nuanced understanding of how stance is conveyed through prosodic cues in oral communication.

In conclusion, this study underscores the complexity of stance-taking in L2 oral discourse and calls for pedagogy that bridges linguistic form, pragmatic function, and cultural nuance. By addressing these gaps, EFL learners can develop a more adaptive repertoire for academic and real-world persuasion.

## Supporting information

S1 Table Stance modals of learners. This table lists the frequency and types of stance modals used by learners.
(XLSX)

S2 Table Stance modals of natives. This table lists the frequency and types of stance modals used by native speakers.
(XLSX)

S1 File README: Annotated Dataset of Stance Modality in Chinese EFL Learners and Native Speakers. This document explains the structure, annotation conventions, and legal/ethical considerations of the dataset files.
(DOCX)

S1 Appendix Appendices. (DOCX)
