# Supplementary material for: Stance modals in Chinese EFL learners’ monologue tasks: A corpus-based study
Source: PLoS One. 2026 Apr 24;21(4):e0347841. doi: 10.1371/journal.pone.0347841 (PMC13108803; doi:10.1371/journal.pone.0347841)
Supplement: S1 File — This document explains the structure, annotation conventions, and legal/ethical considerations of the dataset files. (DOCX) [file pone.0347841.s003.docx]

**README:**

**Annotated Dataset of Stance Modality in Chinese EFL Learners and Native Speakers**

This dataset contains annotated instances of stance modality expressions from spoken English corpora of Chinese EFL learners and native English speakers. The data were drawn from two existing, licensed corpora of spoken English, and consist of task-based monologue performances by Chinese EFL learners and native English speakers. These instances were manually selected and annotated for modality type, stance function, and grammatical features. All data are anonymized and comply with original corpus licensing.

**Dataset Contents**

The dataset is organized into two Excel files:

1. “S1 Table. Stance modals of learners” — data from Chinese EFL learners

2. “S2 Table. Stance modals of natives” — data from native English speakers

Each file contains three sheets representing different types of modality:

1. Sheet 1: Epistemiccity
2. Sheet 2: Deonticity
3. Sheet 3: Potentiality

Each sheet contains annotated instances related to the corresponding modality type.

The structure of each sheet is consistent.

**Column Descriptions**

1. Category: General modality category (i.e., Epistemic modality and Effective modality)
2. Sub-category: furthers groups of the category of Effective modality (i.e., Deontic modality, and Potentiality *can*)
3. Type: Specific stance subtype (e.g., Booster, Hedge, Strong, Weak, ability *can*, possibility *can*, suggestions *can*)
4. Grammatical feature: Linguistic categorization (e.g., “*can* + action verb”)
5. Year/Topic: The year of the speaking test (applicable only to learners) and/or the topic of the speaking task.
6. Proficiency Level (EFL learners only): Speaker’s English level (1 = high; 2 = medium; 3 =low)
7. Speaker: Anonymized speaker ID (e.g., L1_06-004-10 or K_religion)
8. Marker: The target modal word or expression (e.g., *can*, *should*)
9. Marker context: Full sentence or clause from the corpus containing the target expression, with embedded bracketed annotations

**Annotation Conventions in “Marker context”**

Bracketed tags (e.g., <B:M_ability>, <H:M_Possibility>) indicate the identified stance function and modality type. Some tags refer to stance features beyond modality (e.g., grade, address), which are related to other parts of our broader study not covered in this paper. Tag meanings follow an internal annotation scheme defined for the purposes of this study.

Original full transcripts are not included due to licensing constraints.

**Legal and Ethical Notes**

1. No personally identifiable information is included.
2. Speaker IDs have been anonymized.
3. Only annotated excerpts are shared; full transcripts remain under the control of the original corpus owners.
4. The data are provided for academic reuse and transparency per PLOS ONE’s open data policy.
