# Supplementary material for: Stance modals in Chinese EFL learners’ monologue tasks: A corpus-based study
Source: PLoS One. 2026 Apr 24;21(4):e0347841. doi: 10.1371/journal.pone.0347841 (PMC13108803; doi:10.1371/journal.pone.0347841)
Supplement: S1 Appendix — (DOCX) [file pone.0347841.s004.docx]

## Appendix A: Topics for learners’ sub-corpus

| **Year** | **Topic** |
| --- | --- |
| 2003  2004  2005  2006  2007 | Spaceship Columbia: A Final Journey  China’s Employment Market Challenged by More Graduates  Suggestions for the 2008 Beijing Olympics  Should firecrackers and fireworks be allowed during the Spring Festival?  Pets or not? |

## Appendix B: Topics for native speakers’ sub-corpus

| **College Year** | **Topic** |
| --- | --- |
| 1 & 4 | Do you think it is appropriate for college students to rent apartments outside the campus and live there? |
| 2 | Make critical comments on the use of electronic dictionaries among college students. |
| 3 | Do you think it is appropriate for college students to get married? |
